# Beyond apoptosis: evidence of other regulated cell death pathways in the ovary throughout development and life

**DOI:** 10.1093/humupd/dmad005

**Published:** 2023-02-28

**Authors:** Jessica M Stringer, Lauren R Alesi, Amy L Winship, Karla J Hutt

**Affiliations:** Department of Anatomy and Developmental Biology, Monash Biomedicine Discovery Institute, Monash University, Clayton, VIC, Australia; Department of Anatomy and Developmental Biology, Monash Biomedicine Discovery Institute, Monash University, Clayton, VIC, Australia; Department of Anatomy and Developmental Biology, Monash Biomedicine Discovery Institute, Monash University, Clayton, VIC, Australia; Department of Anatomy and Developmental Biology, Monash Biomedicine Discovery Institute, Monash University, Clayton, VIC, Australia

**Keywords:** fertility, ovary, oocyte, granulosa cell, regulated cell death, apoptosis, necroptosis, autophagy, pyroptosis, parthanatos

## Abstract

**BACKGROUND:**

Regulated cell death is a fundamental component of numerous physiological processes; spanning from organogenesis *in utero*, to normal cell turnover during adulthood, as well as the elimination of infected or damaged cells throughout life. Quality control through regulation of cell death pathways is particularly important in the germline, which is responsible for the generation of offspring. Women are born with their entire supply of germ cells, housed in functional units known as follicles. Follicles contain an oocyte, as well as specialized somatic granulosa cells essential for oocyte survival. Follicle loss—via regulated cell death—occurs throughout follicle development and life, and can be accelerated following exposure to various environmental and lifestyle factors. It is thought that the elimination of damaged follicles is necessary to ensure that only the best quality oocytes are available for reproduction.

**OBJECTIVE AND RATIONALE:**

Understanding the precise factors involved in triggering and executing follicle death is crucial to uncovering how follicle endowment is initially determined, as well as how follicle number is maintained throughout puberty, reproductive life, and ovarian ageing in women. Apoptosis is established as essential for ovarian homeostasis at all stages of development and life. However, involvement of other cell death pathways in the ovary is less established. This review aims to summarize the most recent literature on cell death regulators in the ovary, with a particular focus on non-apoptotic pathways and their functions throughout the discrete stages of ovarian development and reproductive life.

**SEARCH METHODS:**

Comprehensive literature searches were carried out using PubMed and Google Scholar for human, animal, and cellular studies published until August 2022 using the following search terms: oogenesis, follicle formation, follicle atresia, oocyte loss, oocyte apoptosis, regulated cell death in the ovary, non-apoptotic cell death in the ovary, premature ovarian insufficiency, primordial follicles, oocyte quality control, granulosa cell death, autophagy in the ovary, autophagy in oocytes, necroptosis in the ovary, necroptosis in oocytes, pyroptosis in the ovary, pyroptosis in oocytes, parthanatos in the ovary, and parthanatos in oocytes.

**OUTCOMES:**

Numerous regulated cell death pathways operate in mammalian cells, including apoptosis, autophagic cell death, necroptosis, and pyroptosis. However, our understanding of the distinct cell death mediators in each ovarian cell type and follicle class across the different stages of life remains the source of ongoing investigation. Here, we highlight recent evidence for the contribution of non-apoptotic pathways to ovarian development and function. In particular, we discuss the involvement of autophagy during follicle formation and the role of autophagic cell death, necroptosis, pyroptosis, and parthanatos during follicle atresia, particularly in response to physiological stressors (e.g. oxidative stress).

**WIDER IMPLICATIONS:**

Improved knowledge of the roles of each regulated cell death pathway in the ovary is vital for understanding ovarian development, as well as maintenance of ovarian function throughout the lifespan. This information is pertinent not only to our understanding of endocrine health, reproductive health, and fertility in women but also to enable identification of novel fertility preservation targets.

## Graphical Abstract

**Graphical Abstract:**
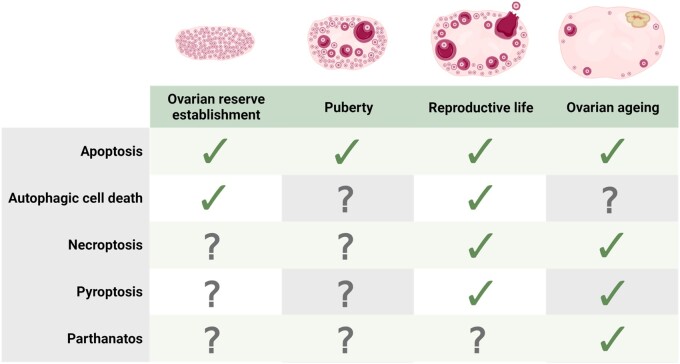
Although apoptosis has well-established roles in regulating ovarian follicle number across the lifespan, recent evidence suggests autophagic cell death, necroptosis, pyroptosis, and parthanatos also contribute.

## Introduction

Regulated cell death plays an integral role in tissue and organ differentiation during foetal development, and in the maintenance of cell turnover throughout subsequent stages of life. These cell death pathways are critical for responding to toxic insults or microbial infection, while dysfunctional cell death signalling can be implicated in the development of some disease states in humans (Kist and Vucic, 2021). Accordingly, some cell death regulators represent viable therapeutic targets. The ovary houses the female germline in the form of oocytes, which are encapsulated by somatic granulosa and theca cells to form ovarian follicles. Women are born with their lifetime supply of follicles termed the ‘ovarian reserve’. Therefore, maintaining the quality of the reserve of long-lived oocytes is crucial to ensure the generation of healthy, viable children (Kerr *et al.*, 2012a).

Across the reproductive lifespan, regulated cell death plays fundamental roles during follicle formation, follicle development, ovulation, and the elimination of damaged oocytes. Therefore, understanding the specific pathways and key factors involved in the regulation of oocyte attrition (death of the oocyte specifically) and follicle atresia (death of the whole follicle, oocyte, and supporting somatic cells) has been the topic of intensive study over the past several decades. Animal models have contributed vital insights since the availability of human tissue for study is limited.

During foetal development, there is a considerable oversupply of female gametes, which are initially generated by foetal germ cell proliferation before entry into meiosis. However, this supply is significantly depleted by extensive oogonial and oocyte loss during follicle formation (Kerr *et al.*, 2013; Findlay *et al.*, 2015). Oogonia and oocyte survival during ovarian development is dependent on the activation or suppression of cell death signals. These signals dictate the size and quality of the follicle pool that women will be endowed with, which will ultimately define their lifetime fertility (Kerr *et al.*, 2013; Findlay *et al.*, 2015). The process of follicle development (folliculogenesis) then commences, during which a subset of immature primordial follicles is activated to develop and mature through discrete stages of growth (pre-antral, antral, and pre-ovulatory), ultimately producing a single mature ovulatory oocyte with each menstrual cycle in women. During puberty, there is a surge in follicle atresia, which is thought to be predominately hormonally induced, though the exact reasons for this remain unknown (Liew *et al.*, 2017). Folliculogenesis occurs throughout reproductive life and, like follicle formation, is subject to a significant amount of redundancy, since more follicles are activated than will actually be ovulated. Indeed, over 99% of activated follicles will undergo natural atresia (Wallace and Kelsey, 2010; Pelosi *et al.*, 2015).

In addition to natural atresia, exposure to endogenous and exogenous insults that may be detrimental to oocyte quality can readily trigger regulated cell death pathways and follicle loss. Although, in some cases, this might be necessary in order to maintain the integrity of the female germline (Winship *et al.*, 2018). For example, it is well-established that exposure to ionizing radiation, certain chemotherapies, and various environmental toxicants can induce DNA damage and significantly alter the balance of follicle atresia versus survival and maturation (Oktem and Oktay, 2007; Tatone *et al.*, 2008; Soleimani *et al.*, 2011; Winship *et al.*, 2018). Additionally—depending on the severity of damage—exposure to such agents can completely ablate the follicle pool, leading to permanent infertility and premature menopause (Kerr *et al.*, 2012a; Titus *et al.*, 2021). Thus, understanding the precise mechanisms of regulated cell death that occur within ovarian follicles in response to various exogenous insults has important implications for developing effective fertility preservation strategies.

In this review, we provide a comprehensive overview of the most recent literature that identifies cell death pathway regulators in the ovary, highlighting apoptotic and non-apoptotic pathways, and their functions throughout the discrete stages of ovarian development and reproductive life.

## Methods

Comprehensive literature searches using PubMed and Google Scholar were conducted for this review to identify peer-reviewed English publications for human, animal, and cellular studies published until August 2022. The search included keywords in the following areas: oogenesis, follicle formation, follicle atresia, oocyte loss, oocyte apoptosis, regulated cell death in the ovary, non-apoptotic cell death in the ovary, premature ovarian insufficiency (POI), primordial follicles, oocyte quality control, granulosa cell death, autophagy in the ovary, autophagy in oocytes, necroptosis in the ovary, necroptosis in oocytes, pyroptosis in the ovary, pyroptosis in oocytes, parthanatos in the ovary, and parthanatos in oocytes.

## Types of regulated cell death that occur within the ovary

**Figure 1.**
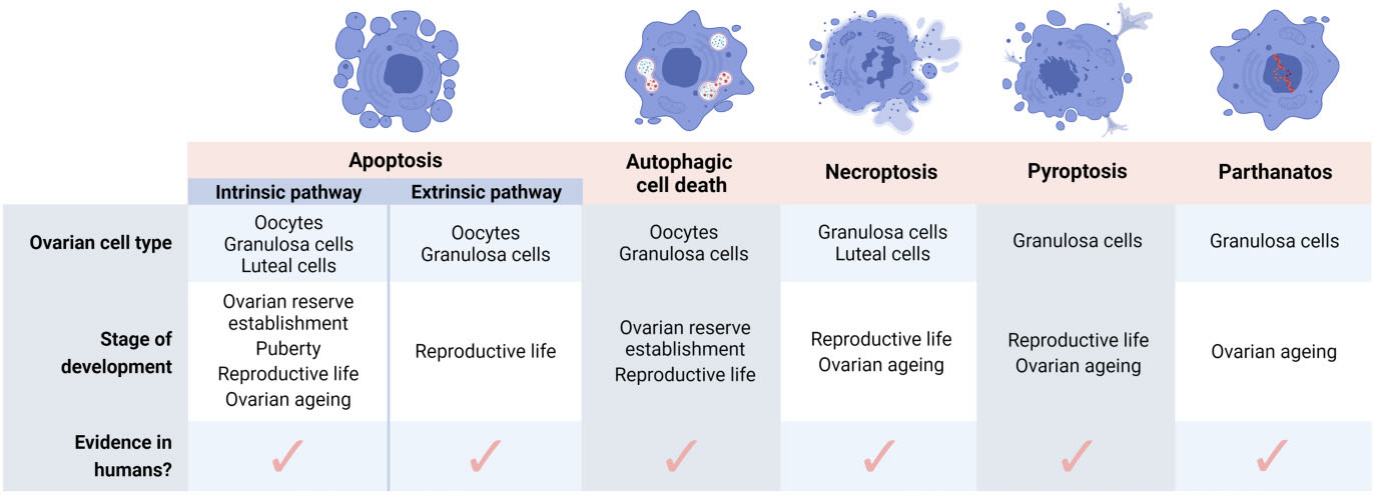
**Types of cell death active within the ovary throughout various stages of development.** The regulated cell death pathways apoptosis (intrinsic and extrinsic), autophagic cell death, necroptosis, pyroptosis, and parthanatos are all active within the ovary in numerous species, including humans. The ovarian cell type and stage of development in which evidence has been published is summarized. Figure created using BioRender.

Regulated cell death refers to the programmed induction of cell death by specific molecular pathways. In the ovary, apoptosis has been the most widely investigated regulated cell death pathway to date (Liew *et al.*, 2016; Marcozzi *et al.*, 2018; Li, 2021), with new insights into the mechanisms of apoptotic cell death within the ovary published regularly (Table I). However, recent evidence indicates that other cell death pathways—including autophagy and autophagic cell death, necroptosis, pyroptosis, and parthanatos—may also play important roles in regulating ovarian function across the lifespan (Fig. 1; Table II). The classification of these various pathways differs in their gross cellular morphology, pathophysiological relevance, and by the specific signal transduction molecules that activate and execute the cell’s demise. However, it is important to appreciate that additional regulated cell death pathways exist in other tissues and cell types (reviewed in detail by Galluzzi *et al.*, 2018).

**Table I:** Recent insights into the mechanisms of apoptotic cell death within the ovary.

Species	Study type	Factor(s)	Developmental stage	Ovarian cell type	Study design	Main findings	Reference
Human	*In vivo*	Leucyl-tRNA synthetase 2 (LARS2)	Follicle atresia	Granulosa cells	Analysis of *LARS2* expression in human granulosa cells derived from patients with premature ovarian insufficiency (POI).	*LARS2* expression is decreased in granulosa cells of POI patients. Knockdown of *LARS2* induces granulosa cell apoptosis and impairs mitochondrial function, by increasing reactive oxygen species (ROS) levels.	(Feng *et al.*, 2022)

		GnRH agonists	Oocyte maturation	Granulosa cells Mature follicles	Analysis of follicular fluid collected from human follicles. Mural granulosa cells and luteal cells isolated from follicular fluid.	Significantly increased apoptosis in cumulus oocyte complexes (COCs) from women treated with GnRH agonists. Suggests that GnRH triggers could impair follicle maturation and trigger corpus luteum regression.	(Gonen *et al.*, 2021)

	*In vitro*	Sirtuin-1 (SIRT1)	Follicle atresia	Granulosa cells	Analysis of primary and immortalized human granulosa cells treated with SRT2104 (a SIRT1 activator).	SRT2104 significantly increased the number of apoptotic cells, as well as elevating pro-apoptotic cleaved caspase-3 and cleaved poly-(ADP-ribose) polymerase (PARP) levels, suggesting SIRT1 is involved in apoptosis within the ovary.	(Sapuleni *et al.*, 2022)

		Anandamide (AEA)	Follicle development Oocyte maturation	Granulosa cells	*In vitro* culture of human immortalized granulosa cells (COV434) and human granulosa cells ± AEA.	AEA reduces cell viability and induces granulosa cell apoptosis via extrinsic pathway. Suggests balance of endocannabinoids is crucial for normal follicle development.	(Costa *et al.*, 2021)

		Phosphate and tensin homolog (PTEN)	Oocyte maturation	Granulosa cells	Analysis of PTEN expression in human granulosa cells. Knockdown of PTEN *in vitro* using shRNA.	PTEN expression promotes granulosa cell apoptosis. Knockdown of PTEN significantly reduces granulosa cell apoptosis.	(Yao *et al.*, 2021)

Cow	*In vitro*	Bone morphogenic protein (BMP) 4	Follicle development	Granulosa cells	Analysis of the expression and function of *BMP4* in cultured bovine cumulus cells.	Knockdown of *BMP4* induced apoptosis and cell-cycle arrest in bovine cumulus cells. BMP4 is an important regulator of granulosa cell proliferation via regulation of apoptosis.	(Tian *et al.*, 2022)

Pig	*In vitro*	Cortisol FSH	Oocyte maturation	Granulosa cells	*In vitro* culture of porcine COCs and granulosa cells ± cortisol and/or FSH.	Cortisol induces granulosa cell apoptosis. FSH prevents this cortisol-induced apoptosis.	(Nakanishi *et al.*, 2021)
Pig		Hypoxanthine (Hx) Growth differentiation factor 9 (GDF9) BMP15	Oocyte maturation	Granulosa cells	*In vitro* analysis of cultured porcine granulosa cells collected from antral follicles.	Hx prevents the G2-M transition in porcine granulosa cells, inducing cell cycle arrest and apoptosis. Oocyte factors GDF9 and BMP15 counteract this effect. Suggests counterbalance of intrafollicular factors is important for regulating cell cycle progression of granulosa cells.	(Li *et al.*, 2020)

Mouse	*In vivo*	Lon protease 1 (LONP1)	Follicle development Follicle atresia	Oocytes	Characterization of oocyte-specific conditional *Lonp1^–/–^* reproductive phenotype in mice.	Conditional loss of *Lonp1* in oocytes significantly depletes primordial and growing follicles, leading to infertility. *Lonp1* is critical for oocyte survival, due to its suppression of apoptosis inducing factor mitochondria-associated 1 (AIFM1) translocation to the nucleus.	(Sheng *et al.*, 2022)

		Complement 1Q-like protein (C1QL1)	Follicle atresia Ovarian ageing	Granulosa cells	Characterization of reproductive phenotype of C1QL1-deficient mice (using C1QL1 antiserum).	Loss of C1QL1 increased granulosa cell apoptosis and antral follicle atresia. C1QL1 has important functions in regulating granulosa cell apoptosis, via AKT/mammalian target of rapamycin (mTOR) signalling.	(Lu *et al.*, 2022)

		Growth hormone Fos and Jun signalling	Ovarian ageing	Follicles Mature oocytes	Treatment of ageing mice with GH. Analysis of follicle counts and superovulation.	GH treatment decreased oocyte apoptosis and improved mature oocyte quality. Suggests decreased GH levels and associated increase in c-Jun N-terminal Kinase (JNK) signalling mediates the age-related decline in oocyte quality.	(Liu *et al.*, 2021)

		TAp63 and ΔNp63 (p63 isoforms)	Ovarian reserve establishment	Primordial follicles	Selective deletion of *Trp63* exon 13 (Δ13p63) in mice, which deletes the TAp63α isoform only.	*Δ13p63^+/–^* mice are completely infertile and have almost complete depletion of primordial follicles by postnatal day (PN) 10 via intrinsic apoptosis. Shows that integrity of the p63 C-terminus is critical for oocyte development and survival.	(Lena *et al.*, 2021)

		Wntless (Wnt pathway regulator)	Reproductive life	Granulosa cells Luteal cells	Characterization of oocyte-and granulosa cell-specific conditional *Wntless^–/–^* reproductive phenotype in mice	Granulosa cell-specific conditional *Wntless^–/–^*mice were subfertile and experienced recurrent miscarriage. Suggests deletion of *Wntless* impairs luteinization and induces granulosa cell apoptosis via intrinsic pathway.	(Cheng *et al.*, 2020)
		Cortisol	Oocyte maturation	Mature oocytes Granulosa cells	Treatment of wild-type and tumour necrosis factor alpha (TNF-α) deficient mice with cortisol *in vivo.*	Cortisol impaired oocyte competence, increased oxidative stress, and induced mural granulosa cell apoptosis via extrinsic pathway.	(Yuan *et al.*, 2020)

		Specificity protein 1 (SP1)	Ovarian reserve establishment	Primordial follicles	Global and granulosa cell-specific *Sp1* knockdown in mice.	Knockdown of *Sp1*, especially in granulosa cells, suppresses nest breakdown, oocyte apoptosis and formation of primordial follicles.	(Cai *et al.*, 2020)

		B lymphoma Mo-MLV insertion region 1 (BMI1)	Ovarian reserve establishment Puberty Reproductive life	Follicles Oocytes Granulosa cells	Characterization of *Bmi^–/–^* mouse reproductive phenotype	Complete infertility, disrupted estrous cyclicity and delayed onset of puberty in *Bmi^–/–^* mice. Significant reduction of primordial follicles and mature oocytes. Increased granulosa cell apoptosis via intrinsic pathway and mitochondrial dysfunction.	(Wang *et al.*, 2019)

	*Ex vivo*	TNF-α BH3 interacting-domain death agonist (BID)	Follicle atresia	Primordial follicles	*Ex vivo* culture of wild-type and *Bid^–/–^* postnatal mouse ovaries with or without TNF-α.	TNF-α significantly depletes primordial follicle numbers in wild-type, but not *Bid^–/–^* ovaries, suggesting that TNF-α can directly induce primordial follicle atresia via the extrinsic apoptosis pathway.	(Winship *et al.*, 2022)

	*In vitro*	Orexin-A (OXA)	Follicle development Follicle atresia	Granulosa cells	Knockdown of OXA receptor 1 (OXR1) in mouse primary granulosa cells.	OXA (a neuropeptide) and OXR1 are expressed in mouse primary granulosa cells. OXA regulates granulosa cell proliferation and apoptosis *in vitro* via the AKT/ERK signalling pathway, thus may have roles in regulating follicle growth and atresia.	(Safdar *et al.*, 2021)

		Chemokine (C–C motif) ligand 5 (CCL5)	Ovarian ageing Reproductive senescence	Granulosa cells	Analysis of cultured mouse ovarian follicles and granulosa cells.	CCL5 impairs oocyte maturation and promotes granulosa cell apoptosis *in vitro*. Suggests CCL5 secretion by theca-interstitial cells may impair follicle development and maturation during ovarian ageing.	(Shen *et al.*, 2019)

*Caenorhabditis elegans*	*In vivo*	DNA topoisomerase 3 (TOP3)	Oocyte quality control and ovarian reserve maintenance	Oocytes	Analysis of *top-3 C. elegans* mutants.	Loss of *top-3* impairs the ability to eliminate defective oocytes, suggesting that *top-3* is critical for oocyte quality control via intrinsic apoptosis.	(Dello Stritto *et al.*, 2021)

**Table II:** Emerging evidence suggesting the contribution of other regulated cell death pathways to ovarian function.

Cell death pathway	Species	Study type	Factor(s)	Developmental stage	Ovarian cell type	Study design	Main findings	Reference
Autophagic cell death	Human	*In vitro*	–	Follicle development Follicle atresia	Granulosa cells	Human granulosa cells treated with apoptosis-inducing substances *in vitro.* Analysis of autophagy and phagocytosis markers.	Granulosa cells ingest and destroy apoptotic oocytes via autophagy-assisted phagocytosis	(Yefimova *et al.*, 2020)

	Cow	*In vitro*	FSH	Oocyte maturation	Granulosa cells	Bovine granulosa cells treated with increasing doses of FSH *in vitro*.	High doses of FSH induce autophagy in bovine granulosa cells. Suggests why aggressive FSH stimulation in patients leads to poor oocyte quality and embryo development.	(Tang *et al.*, 2021a)

	Pig	*In vivo*	Light chain 3B (LC3B)	Follicle atresia Oocyte maturation	Granulosa cells Cumulus cells Oocytes	Analysis of autophagy and apoptosis markers in granulosa cells, cumulus cells, and oocytes isolated from porcine cumulus oocyte complexes (COCs).	Significant increase in abundance of LC3B-II protein in granulosa cells, cumulus cells and oocytes from both early and late stage atretic follicles. Suggests that growing follicle atresia is regulated by both apoptosis and autophagy of granulosa cells.	(Gioia *et al.*, 2019)

	Rat	*In vivo*	Beclin 1 (BECN1)	Follicle atresia	Oocytes	Analysis of BECN1 levels in pre-pubertal, juvenile, and adult rat ovaries.	In atretic oocytes, high levels of BECN1 are coupled with high levels of caspase-3, BAX, and BAK. Suggests that BECN1, a pro-autophagic protein, promotes apoptosis of oocytes.	(Escobar *et al.*, 2019)

		*In vitro* *In vivo*	Hypoxia-inducible factor (HIF)-1α	Corpus luteum formation	Granulosa cells	Rats treated *in vivo* with a HIF-1α inhibitor. *In vitro* analysis of cultured rat granulosa cells.	HIF-1α plays a crucial role in regulating granulosa cell luteinization and subsequent early corpus luteum development. Inhibition of HIF-1α increased apoptosis in early corpora lutea.	(Tang *et al.*, 2021b)

	Mouse	*In vivo*	–	Ovarian reserve establishment	Primordial follicles	Inhibition of autophagy using 3-methyladenine in perinatal mice.	Active autophagy observed in ovaries from 16.5 days post coitum (dpc) to postnatal day (PN) 3. Inhibition of autophagy increased number of cyst oocytes and delayed follicle formation. Suggests autophagy assists in germ cell cyst breakdown and primordial follicle assembly.	(Zhihan *et al.*, 2019)

		*Ex vivo*	Lysine-specific demethylase 1 (LSD1)	Ovarian reserve establishment	Primordial follicles	*Ex vivo* culture of perinatal mouse ovaries, with *Lsd1* either knocked down or overexpressed using specific siRNAs.	LSD1 is highly expressed in mouse foetal ovaries, but sharply reduces from 18.5 dpc onwards. Suggests that, via regulation of autophagy, LSD1 contributes to the initiation of apoptosis during ovarian reserve establishment.	(He *et al.*, 2020)
Necroptosis	Human	*In vivo*	Phosphoglycerate translocase 5 (PGAM5)	Ovarian ageing	Granulosa cells	Analysis of PGAM5 expression in human cumulus cells.	PGAM5 expression in human cumulus cells increases with advancing age and is associated with decreased mitochondrial function, which may implicate a role for necroptosis in the process of ovarian ageing.	(Li *et al.*, 2022)

		*In vitro*	Sirtuin-1 (SIRT1)	Follicle atresia	Granulosa cells	Analysis of primary and immortalized human granulosa cells treated with SRT2104 (a SIRT1 activator) and Nec-1 (a necroptosis inhibitor).	SRT2104 significantly increased the number of necrotic cells, as well as elevating pro-necroptotic receptor-interacting serine/threonine-protein kinase (RIPK) 1 and mixed lineage kinase domain-like pseudokinase (MLKL) protein levels. Nec-1 attenuated RIPK1 and MLKL levels, suggesting SIRT1 is involved in necroptosis within the ovary.	(Sapuleni *et al.*, 2022)

	Cow	*In vivo*	RIPK1 and RIPK3	Oocyte maturation	Granulosa cells	Analysis of mRNA expression of RIPK1 and RIPK3 in granulosa and theca cells derived from healthy and atretic bovine follicles.	Suggests that both apoptosis and necroptosis occur within granulosa cells of dominant follicles undergoing luteinization.	(McEvoy *et al.*, 2021)

	Pig	*In vivo*	Chemerin	Corpus luteum regression	Luteal cells	High throughput sequencing of the transcriptome of cultured mid-luteal stage porcine luteal cells.	Chemerin (an adipokine) interacts strongly with necroptosis-associated genes during the mid-luteal phase, suggesting a potential role for necroptosis (in conjunction with apoptosis) in facilitating corpus luteum regression.	(Makowczenko *et al.*, 2022)

Pyroptosis	Human	*In vivo*	Gasdermin family members (GSDMs)	–	–	Analysis of expression of GSDMs in human ovarian tissue from patients with serous ovarian cancer and healthy counterparts.	Many GSDMs, including gasdermin D (GSDMD—a key pore-forming protein involved in pyroptosis) are expressed in normal ovarian tissue.	(Berkel and Cacan, 2021)

	Cow	*In vivo* *In vitro*	Non-esterified fatty acids (NEFAs)	Follicle atresia	Granulosa cells	Analysis of serum and cultured granulosa cells obtained from bovine ovaries.	NEFAs induced pyroptosis and inflammation of granulosa cells *in vitro*, as evidenced by increased NLR family pyrin domain containing 3 (NLRP3), toll-like receptor 4 (TLR4), caspase-1, and interleukin-1β expression.	(Wang *et al.*, 2020)
	Rat	*In vivo* *In vitro*	Polystyrene microplastics (PS MPs)	Follicle atresia	Granulosa cells	Analysis of serum, ovaries and cultured primary granulosa cells in rats.	PS MPs activated the NLRP3/caspase-1 signalling pathway in ovarian granulosa cells possibly triggered by oxidative stress.	(Hou *et al.*, 2021)

	Mouse	*In vivo*	NLRP3 inflammasome	Ovarian ageing	Follicles	Characterization of *Nlrp3^–/–^* mouse phenotype across the reproductive lifespan.	Although not directly assessed, both publications suggest that pyroptosis is contributing to age-related follicle depletion in mice, via activation of the NLRP3 inflammasome.	(Lliberos *et al.*, 2020) (Navarro-Pando *et al.*, 2021)

Parthanatos	Human	*In vitro*	Poly(ADP-ribose) (PAR)	Oocyte maturation	Cumulus granulosa cells	Oocytes collected from normal and diminished ovarian reserve (DOR) patients. Cumulus cells isolated and cultured.	Increased PAR expression in cumulus cells of DOR patients. Suggests poly[ADP-ribose] (PAR) polymerase 1 (PARP-1)-dependent cell death may contribute to diminished ovarian reserve.	(Batnasan *et al.*, 2020)

### Apoptosis

**Figure 2.**
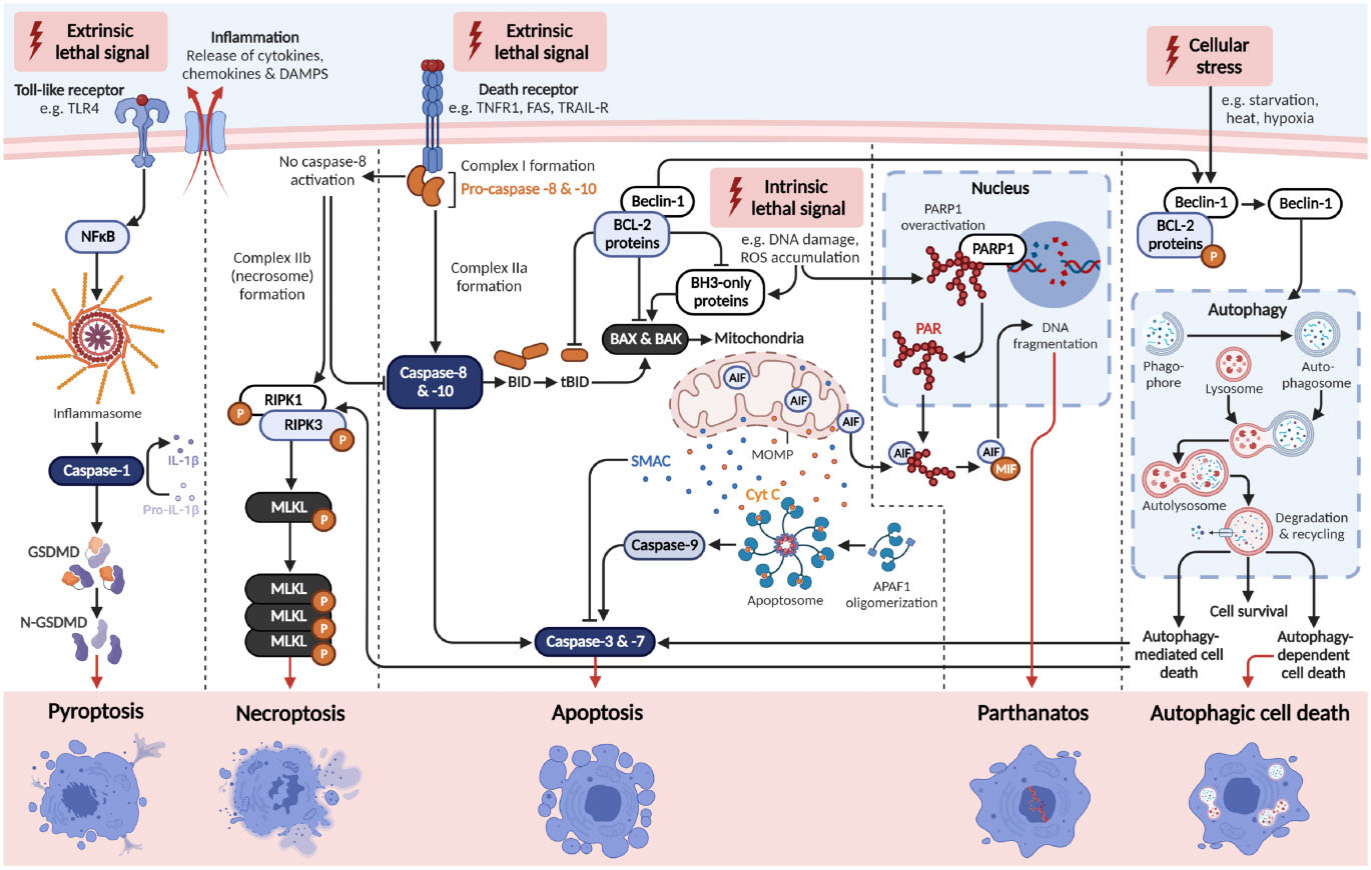
**Overview of regulated cell death pathways.** A summary of each of the well-described regulated cell death pathways—apoptosis (intrinsic and extrinsic), autophagic cell death, necroptosis, pyroptosis, and parthanatos. **Intrinsic apoptosis:** After an intrinsic lethal signal occurs (e.g. DNA damage), BH3-only proteins activate BAX and BAK either directly, or indirectly by binding and inhibiting BCL-2 proteins. Mitochondrial outer membrane permeabilization (MOMP) then occurs, which releases cytochrome C (Cyt C) and SMAC, the latter of which can inhibit apoptosis. The apoptosome is then formed, leading to caspase-9 activation, subsequent caspase-3 and -7 activation, and initiation of apoptosis. **Extrinsic apoptosis**: Once death receptors (e.g. TNFR1, FAS, or TRAIL-R) detect an extrinsic lethal signal, this receptor associates with pro-caspase-8 and -10 to form complex I. Complex IIa is subsequently formed, which leads to caspase-8 and -10 activation. Apoptosis is then initiated either directly, via direct cleavage of caspase-3 and -7; or indirectly, via cleavage of BID into tBID and subsequent activation of BAX and BAK. **Necroptosis:** Following an extrinsic lethal signal and in the absence of caspase-8 activation, complex IIb (i.e. the necrosome) is formed. This leads to phosphorylation of receptor-interacting serine/threonine-protein kinase (RIPK) 1 and 3, which phosphorylate and activate mixed lineage kinase domain-like pseudokinase (MLKL). MLKL then forms a complex, resulting in release of cytokines, chemokines, and damage-associated molecular patterns (DAMPS). Ultimately, this results in inflammation and necroptosis of the cell. **Pyroptosis:** Once toll-like receptors (e.g. TLR4) detect an extrinsic lethal signal, nuclear factor kappa B (NF-κB) signalling is activated. This results in inflammasome formation and subsequent caspase-1 activation. Then, pro-IL-1β is converted into IL-1β, and gasdermin D (GSDMD) is cleaved into N-GSDMD fragments. This leads to inflammation and pyroptosis of the cell. **Parthanatos:** Once an intrinsic lethal signal occurs (e.g. excessive reactive oxygen species accumulation), poly[ADP-ribose] polymerase 1 (PARP-1) becomes activated. If PARP-1 overactivation occurs, this can lead to accumulation of PAR polymer and translocation of apoptosis inhibitory factor (AIF) from mitochondria. AIF forms a complex with macrophage migration inhibitory factor (MIF), which re-enters the nucleus. Ultimately, this leads to DNA fragmentation and parthanatos of the cell. **Autophagic cell death:** Beclin-1 normally exists in a complex with BCL-2 proteins. Once these have been phosphorylated and inactivated, free Beclin-1 can then initiate autophagy. Autophagy involves fusion of the autophagosome and lysosome to form the autolysosome, which then degrades and recycles intracellular components. This can lead to cell survival, but sometimes can cause autophagy-mediated cell death (by activating either apoptosis or necroptosis) or autophagy-dependent cell death (i.e. cell death without apoptosis or necroptosis). Figure created using BioRender.

Apoptosis is the programmed, controlled death of a cell; which involves degradation and fragmentation of protein and DNA, and engulfment of the collapsed cell by neighbouring cells and/or phagocytes in a non-inflammatory manner. It occurs in all multicellular organisms throughout life from foetal development onwards and is an essential homeostatic mechanism to maintain healthy cell populations in tissues and organs. There are two major apoptotic pathways: the intrinsic and extrinsic pathways (Fig. 2). The intrinsic (mitochondrial) pathway is activated from within the cell and is predominately regulated by mitochondria. On the other hand, the extrinsic (death receptor) pathway is triggered from outside the cell, typically in response to conditions and factors within the extracellular environment.

#### Intrinsic apoptosis

The intrinsic apoptosis pathway is initiated by a variety of non-receptor-mediated microenvironmental perturbations, including growth factor withdrawal, DNA damage, endoplasmic reticulum stress, reactive oxygen species (ROS) overload, replication stress, and microtubular alterations or mitotic defects, among others (Elmore, 2007; Li and Yuan, 2008; Suen *et al.*, 2008; Wang and Youle, 2009). These stimuli produce intracellular signals that cause disruptions to the mitochondrial membrane, which result in mitochondrial inner and outer membrane permeabilization, loss of mitochondrial transmembrane potential, and release of normally sequestered pro-apoptotic proteins from the intermembrane space into the cytosol. This release of pro-apoptotic proteins is considered the ‘point of no return’ in apoptosis, after which cytochrome c release, caspase activation (predominately caspase-3), formation of the apoptosome, and death of the cell will occur (Aubrey *et al.*, 2018).

The intrinsic apoptosis pathway is controlled by members of the B cell lymphoma 2 (BCL-2) family of proteins. These can be divided into three subgroups based on their structure and function: the anti-apoptotic BCL-2 proteins, including BCL-2, BCL-XL, BCL-W, MCL-1, and A1; the pro-apoptotic proteins, BAX, BAK, and BOK; and the BH3-only proteins PUMA, NOXA, BH3 interacting-domain death agonist (BID), BAD, BIM, BIK, HRK, and BCL-2 modifying factor (BMF). The BH3-only proteins are responsible for sensing apoptotic signals and transmitting them to other BCL-2 family members to ultimately trigger the apoptotic cascade. They do this by binding and inhibiting the core anti-apoptotic BCL-2 proteins, leading to conversion of BAX, BAK, and BOK from inert monomers into membrane-permeabilizing oligomers (Moldoveanu and Czabotar, 2020). Once permeabilized, apoptogenic factors, such as cytochrome c, are released from the mitochondrial intermembrane space and trigger caspase activation.

#### Extrinsic apoptosis

The extrinsic apoptotic pathway can be activated by two types of plasma membrane receptors: death receptors, which are activated by cognate ligand binding (Guicciardi and Gores, 2009); and dependence receptors, which are activated when specific ligands drop below a certain threshold (Goldschneider and Mehlen, 2010). The most widely characterized death receptors include, but are not limited to, FAS cell surface death receptors and the tumour necrosis factor (TNF) receptor superfamily members (Wajant and Siegmund, 2019). Briefly, binding of the death receptor ligand to the receptor allows the assembly of the death-inducing signalling complex that regulates the activation of pro-caspase-8 and -10.

Although the mitochondrial pathway is strongly associated with intrinsic apoptosis; in certain cell types, the extrinsic pathway can crosstalk with the intrinsic pathway through caspase-8-mediated proteolytic cleavage of _t_BID to BID, which triggers the release of apoptogenic factors to activate BAX and induce apoptosis (Cui *et al.*, 2016).

### Autophagy and autophagic cell death

Derived from the Greek language, meaning ‘self-eating’, autophagy is a tightly-regulated process whereby cells degrade and recycle their own cytosolic components inside lysosomes, which can lead to cell death (Galluzzi *et al.*, 2018; Schwartz, 2021). Unlike apoptosis, this form of regulated cell death occurs in the absence of chromatin condensation and phagocytes. Autophagic cell death manifests in the accumulation of large numbers of autophagic vesicles containing cytoplasmic material for degradation by lysosomes, and results in early degradation of organelles and late degradation of cytoskeleton, which is the reverse for apoptotic cells (Cuervo, 2004; Thorburn, 2008).

Autophagy is mediated by dozens of autophagy-related (ATG) proteins that regulate expanding ‘isolation membranes’, which encapsulate and enclose proteins/organelles into a double-membrane structure called the autophagosome (Li *et al.*, 2021b). These autophagosomes then fuse with liposomes to degrade the internal components. Once fused, acidic hydrolases in the lysosome can degrade the autophagic cargos, and salvaged nutrients are released to the cytoplasm to be recycled by cells. Genetic models have demonstrated that some autophagic machinery is essential for regulated cell death (e.g. ATG1, reviewed by Schwartz, 2021); however, it has been suggested that it might be more appropriate to name the process ‘autophagy-mediated cell death’ (Kroemer and Levine, 2008). Although, more recent literature suggests that autophagy-dependent cell death, that is independent of apoptosis or other regulated cell death pathways, can occur (Bialik *et al.*, 2018; Denton and Kumar, 2019; Kriel and Loos, 2019) (Fig. 2). Indeed, there is strong evidence that apoptotic and autophagic machineries are highly interconnected during developmental regulated cell death (Zhang and Baehrecke, 2015).

### Necroptosis

Necrosis is morphologically distinct from apoptosis and characterized by a gain in cell volume, organelle swelling, plasma membrane rupture, and loss of intracellular contents (Galluzzi *et al.*, 2018). Unlike apoptosis, necrosis provokes an inflammatory response by spilling the cell’s cytosolic constituents into the extracellular space through the damaged plasma membrane. During apoptosis, however, these products are safely isolated by membranes and then consumed by phagocytes. Necroptosis is a programmed, regulated form of necrosis that is initiated by various extracellular and intracellular stressors, including viral infection (Guo *et al.*, 2018), inflammation (Pasparakis and Vandenabeele, 2015), and factors detected by specific death receptors (e.g. FAS, TNFR1) or pathogen recognition receptors (Galluzzi *et al.*, 2018; Frank and Vince, 2019) (Fig. 2). Importantly, these death receptors can also activate the extrinsic apoptosis pathway (Grootjans *et al.*, 2017; Frank and Vince, 2019). At the molecular level, necroptosis critically depends on activation of the receptor-interacting serine/threonine-protein kinase (RIPK) 1/3 necrosome and mixed lineage kinase domain-like pseudokinase (MLKL), in the absence of caspase-8 activation, to ultimately cause cell membrane rupture (Galluzzi *et al.*, 2018). Indeed, necroptosis is generally observed as a fall-back regulated cell death mechanism that is triggered when apoptosis is hindered, such as during pathogen infection (Brault and Oberst, 2017; Naderer and Fulcher, 2018).

### Pyroptosis

Exogenous insults also extend to infection, and regulated cell death is proposed to contribute to immune defence against infections (Jorgensen *et al.*, 2017). Pyroptosis is mediated by the cleavage of gasdermins, caspases (namely caspase-1), or granzymes; leading to the formation of pores in the cell membrane, lysis of the cell, and the release of inflammatory molecules (Frank and Vince, 2019) (Fig. 2). This type of regulated cell death is primarily observed in inflammatory cells, such as macrophages, and occurs most frequently upon infection with intracellular pathogens (Xia *et al.*, 2019). As such, it is likely to form part of the host response to control bacterial, viral, fungal, or protozoan pathogens (Xia *et al.*, 2019). While the sterile inflammatory response is required for organ development and tissue repair, dysregulation of this process may lead to inflammatory disease, for example asthma, Type 2 diabetes, and inflammatory liver diseases (Rock *et al.*, 2010). Indeed, there is accumulating evidence that pyroptosis and inflammasome dysregulation may contribute to sterile inflammatory diseases, gynaecological diseases, autoimmune diseases, neuronal diseases, and even cancer (Li *et al.*, 2021a; Yu *et al.*, 2021). Moreover, cytokine dysregulation resulting in a pre-inflammatory phenotype that occurs with age, known as ‘inflammageing’ (Rea *et al.*, 2018), has also been associated with pyroptosis (Mejias *et al.*, 2018).

### Parthanatos

Parthanatos is a poly[ADP-ribose] polymerase 1 (PARP1)-dependent and apoptosis-inducing factor (AIF)-mediated, caspase-independent cell death pathway, which is distinct from apoptosis, necroptosis, or other known forms of regulated cell death (Fig. 2). Parthanatos is associated with various diseases including several retinal diseases, Parkinson’s disease, stroke, heart attack, and diabetes (David *et al.*, 2009). Parthanatos is triggered by an excessive ROS response, which leads to an accumulation of poly[ADP-ribose] (PAR) polymer and translocation of AIF from mitochondria to the nucleus, resulting in chromatin condensation and nuclear fragmentation (David *et al.*, 2009; Wang *et al.*, 2011). Parthanatos does have shared characteristics with necroptosis, including loss of membrane integrity and depletion of cellular energy stores (NAD and ATP) (Yu *et al.*, 2003). However, cells undergoing parthanatos experience regulated chromatolysis without swelling and rupturing of cell membranes (Andrabi *et al.*, 2008), as occurs during necroptosis. Key morphological features of parthanatos include shrunken, condensed nuclei, and membrane disintegration (Andrabi *et al.*, 2008).

## Timing and pathways of regulated cell death in ovarian development and function

Regulation of cell death pathways is critical to orchestrating numerous aspects of ovarian development and function across the lifespan (Fig. 1). More than 99% of mammalian follicles will not reach ovulation, instead undergoing atresia (Wallace and Kelsey, 2010; Pelosi *et al.*, 2015). It is well-established that follicle atresia is predominately mediated by apoptosis. In women, germ cell number peaks at ∼5 months gestation, with ∼6.8 million germ cells present in the ovary. This number falls to ∼1 million at birth, and by the onset of puberty, the follicle pool contains only 300 000 follicles (Baker, 1963). Despite this, over a woman’s reproductive life, only ∼400 oocytes will survive and undergo maturation to ovulation (Morita and Tilly, 1999). At ∼50 years of age, ovarian senescence and menopause is triggered when a critical threshold of <1000 follicles remain in the ovary (Faddy *et al.*, 1992).

**Figure 3.**
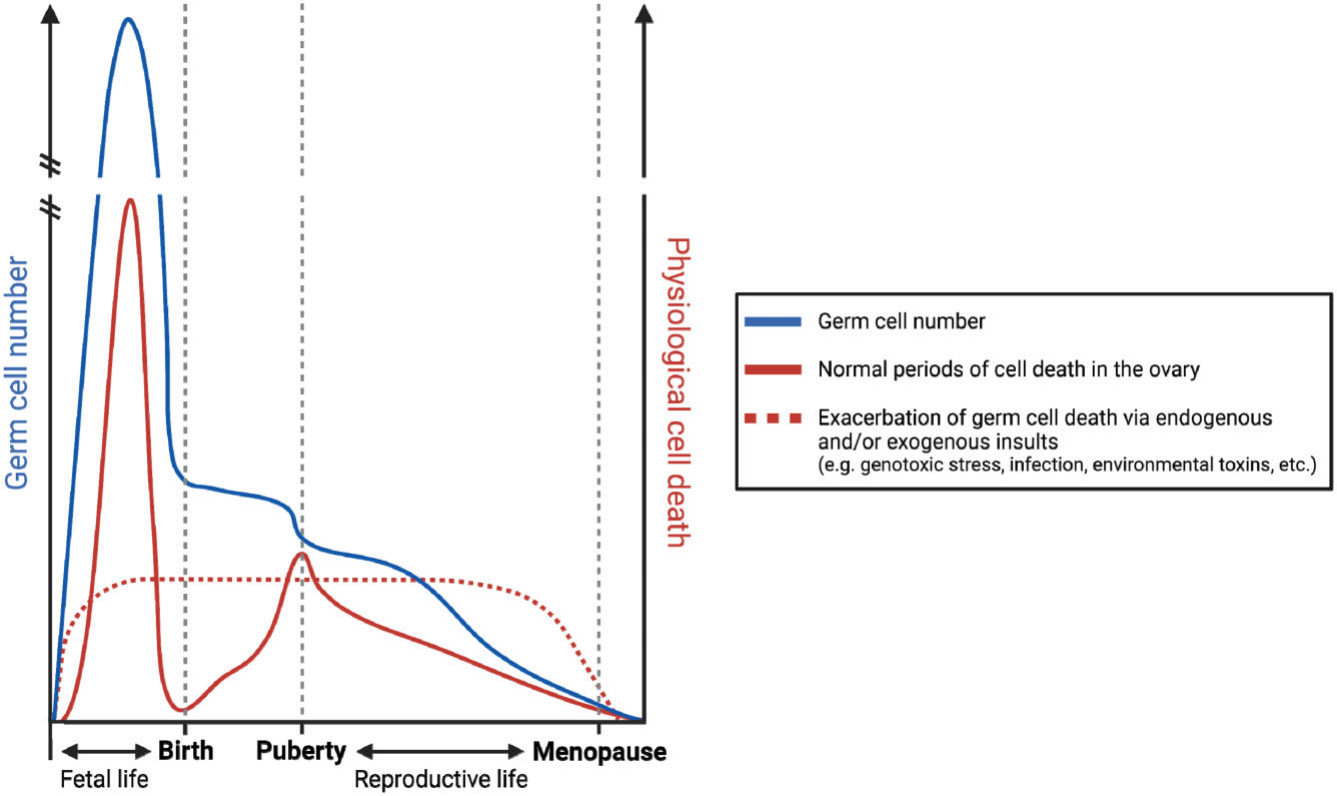
**Timing of regulated cell death across the lifespan within the ovary in women.** It is well-defined that multiple windows of increased germ cell loss across the female lifespan. These include immediately prior to birth and puberty. After puberty, there is a consistent decline in germ cell number across reproductive life, until menopause ensues. However, spikes in loss can be induced in response to endogenous and/or exogenous insults. Figure created using BioRender.

Whilst there is continuous loss of germ cells throughout life, there are two distinct waves during which large numbers of oocytes and primordial follicles are lost in a short period of time (Fig. 3). The first occurs prior to birth in humans, during which primordial follicle formation is occurring, and a second occurs at the onset of puberty. It is now widely accepted that granulosa cell apoptosis is primarily responsible for the atresia of growing follicles, whereas primordial follicle atresia is largely initiated by oocyte apoptosis (Vaskivuo and Tapanainen, 2003; Meng *et al.*, 2018; Regan *et al.*, 2018). However, alternative regulated cell death pathways can also trigger follicle atresia across the lifespan, especially following exposure to certain environmental stressors and toxicants.

### Ovarian reserve establishment

Germ cell loss occurs throughout the process of primordial follicle formation, predominately via apoptosis (McClellan *et al.*, 2003). However, our analysis of studies performed in mice reveals some discrepancies in the exact timing of apoptosis. One of the earliest characterizations was by Bakken and McClanahan (1978), who identified germ cell degeneration (death) by their condensed nuclei containing rounded clumps of densely stained chromatin. This study reported increased germ cell degeneration during the last mitotic divisions, as oocytes become arrested in the first meiotic prophase (Bakken and McClanahan, 1978). At this time, germ cell nests begin to break down and facilitate primordial follicle assembly, culminating in loss of up to 98% of germ cells (Pepling and Spradling, 2001). Data examining germ cell loss during the early stages of meiosis (embryonic day 13.5–17.5 in mice; approximately mid-gestation in humans) are variable. Some reports highlight little to no germ cell loss and few TUNEL-positive-stained germ cells at this time (Pepling and Spradling, 2001). Meanwhile, others have identified a continuous decline and increased proportion of apoptotic germ cells during the first meiotic prophase compared to oogonia undergoing mitosis (Coucouvanis *et al.*, 1993; McClellan *et al.*, 2003). Thus, it is still debated by the field as to whether there are specific windows of germ cell loss during foetal development, or if this loss occurs continuously in the lead up to nest breakdown and follicle formation. Irrespective of the timing of loss, it is well-established that the key anti-apoptotic proteins (e.g. BCL-XL and BCL-2) and pro-apoptotic proteins (e.g. BAX, BMF, and PUMA) are clearly required for this process (Rucker *et al.*, 2000; Flaws *et al.*, 2006; Ke *et al.*, 2013; Myers *et al.*, 2014; Vaithiyanathan *et al.*, 2016).

Extensive germ cell loss occurs during nest breakdown. It has been proposed that these dying cells could be acting as nurse-like cells that transfer important organelles (including centrioles, Golgi, and mitochondria) and the cytoplasm to a select few oogonia, before undergoing regulated cell death (Lei and Spradling, 2016). The remaining oogonia that acquired these additional cellular components become progressively larger, mature, and form the primordial follicle pool. Apoptosis is fundamental to orchestrating nest breakdown and primordial follicle assembly (Lei and Spradling, 2016). However, autophagy likely also plays a key role in this process (recently reviewed by Bhardwaj *et al.*, 2022). Interestingly, inhibiting autophagy mediators using 3-methyladenine delays follicle formation and results in an increased number of cyst oocytes (Rodrigues *et al.*, 2009; Zhihan *et al.*, 2019). In support, germ cell survival immediately prior to and during follicle formation is severely impaired in mice with genetic loss of autophagy-related genes *Atg7* and *Becn1* (Gawriluk *et al.*, 2011; Song *et al.*, 2015). Lysosome amplification—a hallmark of autophagy—is also observed in oocytes upon birth and is most apparent in primordial follicle oocytes (Rodrigues *et al.*, 2009). Lysine-specific demethylase 1 (LSD1)—a critical repressor of autophagy—is highly expressed in mouse foetal ovaries, but sharply reduces during the period of follicle assembly. Expression data, together with functional studies *in vitro*, indicate that LSD1 is an indispensable regulator of oocyte death during ovarian reserve establishment, via regulation of autophagy (He *et al.*, 2020). During the foetal-to-neonatal transition in mammals, which is associated with a transformation in nutrient supply from the maternal–foetal blood interface to lactation, there is a disruption in nutrient supply to the ovary (Kuma *et al.*, 2004). Interestingly, autophagy has been shown to play a critical role in nutrient stress adaptation to prevent excessive germ cell loss during this period in mice (Song *et al.*, 2015; Sun *et al.*, 2018). Together, these studies imply key roles for autophagy mediators in regulating oocyte survival, particularly during nest breakdown and primordial follicle formation.

Once established, primordial follicles enter meiotic arrest. Deemed ‘non-growing follicles’, the oocytes remain arrested at the diplotene stage of meiotic prophase 1, and the surrounding granulosa cells have low mitotic potential (Hartshorne *et al.*, 2009). These primordial follicles represent the stockpile of oocytes available to females for their reproductive life. As such, these primordial follicle oocytes are some of the longest living cells in the mammalian body, and may remain arrested for decades in humans (Pelosi *et al.*, 2015). These follicles will ultimately leave this period of dormancy, either by activation to continue through stepwise maturation via follicle development, or to undergo follicle atresia and oocyte loss. Regulation of this process and a balance between growth, survival, and atretic factors are essential in maintenance of normal reproductive function.

### Puberty

During puberty, rising gonadotrophin levels cause dynamic physiological changes in the ovary, including the development of antral follicles to the Graafian (pre-ovulatory) stage and the onset of ovulation. Strikingly, this window also coincides with a significant spike in the loss of the ovarian reserve of primordial follicles, by approximately half during the adolescent/young adult period (ages 13–25 years) in humans (Wallace and Kelsey, 2010) and roughly two-thirds in mice (Allan *et al.*, 2006; Bristol-Gould *et al.*, 2006). This loss is thought to be gonadotrophin-mediated, though the precise mechanisms remain unknown (Liew *et al.*, 2017). Notably, overexpression of LH in juvenile mice has been shown to trigger depletion of the primordial follicle reserve (Flaws *et al.*, 1997). Conversely, LH and FSH suppression prevented follicle loss in a more recent study (Liew *et al.*, 2017). Importantly, this primordial follicle loss is regulated by apoptosis, as demonstrated by the essential requirement for the pro-apoptotic BH3-only protein, BMF, in this process (Liew *et al.*, 2017).

It remains unclear why such vast numbers of primordial follicles are eliminated at the time of sexual maturation. One explanation for this may be the fact that there are two separate populations of primordial follicles in the ovary that each have distinct functional roles. Class 1 primordial follicles, which are localized to the ovarian medulla, will activate and grow during pre-pubertal life but never be ovulated. These are fast growing, taking 19–21 days to reach maturity in mice (Gilchrist *et al.*, 2001). They will contribute to the first wave of follicle activation, which is in turn likely required for the establishment of the hypothalamic–pituitary–ovarian axis, and puberty onset. Class 2 primordial follicles, located at the ovarian cortex, are slower growing (∼47 days) and are thought to represent the source of all mature ovulatory oocytes for fertilization (Mork *et al.*, 2012; Zheng *et al.*, 2014). Therefore, it is possible that the spike in oocyte loss observed at the transition of puberty involves the clearance of any remaining Class 1 primordial follicles in the medulla. While the two classes of follicles have not been clearly identified in human ovaries, the growth pattern of the first wave of activated follicles during foetal development is conserved in humans (Lintern-Moore *et al.*, 1974; Peters *et al.*, 1975). Moreover, in human ovaries, nearly 20%, 5%, and 0% of the primordial follicles in pre-pubertal, pubertal, and adult ovaries, respectively, are classed as morphologically abnormal (Anderson *et al.*, 2014). Cultures of human cortical tissue containing primordial follicles and isolated pre-antral follicles from pre-pubertal and pubertal girls exhibited low activation rates and compromized oocyte growth respectively, compared to adult samples (Anderson *et al.*, 2014). Thus, it can be speculated that these abnormal primordial follicles are either eliminated or preferentially activated before puberty onset and the commencement of ovulation in humans (Anderson *et al.*, 2014). This provides an intriguing parallel to the two distinct types of primordial follicles identified by Mork *et al.* (2012) in mice (Anderson *et al.*, 2014).

### Follicle atresia throughout reproductive life

Atresia is a complex process that naturally occurs to regulate the follicle pool across the lifespan (Regan *et al.*, 2018). It affects all stages of follicular development and involves multiple forms of regulated cell death. The highest incidence of follicular degeneration is observed when follicles become dependent on FSH, at the early antral follicle stage (Chun *et al.*, 1996). Atresia is essential for maintaining ovarian homeostasis, and the dysregulation of atresia contributes to reproductive disorders, including PCOS and POI (Duncan, 2014).

#### Apoptosis

The regulation of intrinsic apoptosis ensures the maintenance of the number and the quality of the long-lived primordial follicle pool. This pathway is responsible for eliminating defective oocytes, which is paramount to sustain fertility and generate healthy offspring. Genotoxic stress (i.e. DNA damage) within oocytes—particularly in the form of double-stranded breaks—is extremely harmful to chromosome structure and overall DNA integrity. DNA damage can readily accumulate within primordial follicle oocytes as a consequence of normal cellular metabolism and increased levels of oxidative stress during the ageing process (Titus *et al.*, 2013; Stringer *et al.*, 2018; Winship *et al.*, 2018). Additionally, DNA damage can be induced exogenously following exposure to various exogenous insults, including ionizing radiation, environmental toxicants, and certain chemotherapies causing extensive primordial oocyte apoptosis (Oktem and Oktay, 2007; Tatone *et al.*, 2008; Soleimani *et al.*, 2011; Winship *et al.*, 2018).

In oocytes, intrinsic apoptosis is predominately regulated by TAp63α—an isoform of p63, which is the major p53 family member present. TAp63α, and its downstream effector PUMA, are primarily responsible for initiating oocyte apoptosis in response to genotoxic stress *in vivo* (Suh *et al.*, 2006; Livera *et al.*, 2008; Kerr *et al.*, 2012b). Building on this work, a recent report showed that the ovaries in neonatal mice with a *Trp63* exon 13 deletion (which leads to selective silencing of the TAp63α, but not the β isoform) were almost completely devoid of oocytes (Lena *et al.*, 2021). This phenotype was a consequence of increased transcription of *Puma* and *Noxa* expression cause by the constitutively active TAp63β isoform. These data reveal that control of p63 signalling, and the intrinsic apoptosis pathway, is fundamentally important for oocyte maintenance.

Antral follicular degeneration is predominantly initiated by granulosa cell apoptosis. Activation of the death ligand–receptor system is the most common trigger of granulosa cell apoptosis, via the extrinsic apoptosis pathway specifically (Inoue *et al.*, 2011; Chu *et al.*, 2018). The FAS–FAS ligand (FAS-L) system has been localized to the human and rodent ovary, primarily in the granulosa and theca cells of unhealthy pre-antral and antral follicles, and in luteal cells of the corpus luteum (Albamonte *et al.*, 2019), indicating a role in ovarian follicular atresia and luteolysis. Indeed, co-culture of interferon-γ pre-treated granulosa cells and denuded oocytes resulted in granulosa cell apoptosis, which could be blocked by an inhibitor of the FAS–FAS-L interaction (Hakuno *et al.*, 1996). Additionally, new evidence suggests that the extrinsic apoptosis pathway may also regulate atresia of primordial follicles. A recent study examining the impact of checkpoint inhibitor immunotherapy on ovarian function in a mouse model revealed that TNF-α can directly induce primordial follicle loss via BID, which is a key member of the extrinsic apoptosis pathway (Winship *et al.*, 2022).

#### Autophagy and autophagic cell death

Emerging evidence suggests that autophagic cell death plays a role in follicle atresia (recently reviewed by Bhardwaj *et al.*, 2022). Granulosa cell apoptosis may also be triggered by the accumulation of autophagic vacuoles (autophagosomes) leading to the down-regulation of BCL-2 expression (Choi *et al.*, 2010, 2011), suggesting that autophagy is closely related to apoptosis induction in granulosa cells. Interestingly, recent investigations show that different regulated cell death pathways beyond apoptosis play active roles in mediating follicle atresia, depending on the stage of follicle development. Pre-antral follicle atresia occurs largely via enhanced granulosa cell autophagy, meanwhile antral follicle atresia arises due to granulosa cell apoptosis (Meng *et al.*, 2018). The oocyte residing within the atretic follicle may then be eliminated by mechanisms involving mediators common to both apoptosis and autophagy pathways (Escobar *et al.*, 2008; Sanchez *et al.*, 2012; Escobar *et al.*, 2019). Unlike standard morphological features of apoptosis, oocytes within atretic follicles do not display normal chromatin compaction; however, they do display DNA fragmentation. Therefore, oocyte death may begin with autophagic degradation of cytoplasmic components, including mitochondria, which activates caspases that lead to DNA fragmentation without compaction; thus, triggering a non-conventional route of cell death.

There is, in fact, evidence that autophagy contributes to the regulation of oocyte death and follicle atresia at all stages of development, though its role in cell survival versus cell death appears to be complex. The expression of autophagy-related genes (e.g. those encoding ATG proteins, microtubule-associated proteins 1A/1B light chain 3 A and B [LC3A/B], beclin-1 [BECN1] and [LAMPs]) have been detected in follicles at all stages of development in rodents and pigs (Rodrigues *et al.*, 2009; Choi *et al.*, 2010; Gawriluk *et al.*, 2011; Hale *et al.*, 2017; Ullah *et al.*, 2019; Leopardo *et al.*, 2020). In mice, the relative mRNA expression of *Becn1* is highest in primordial follicle oocytes compared with oocytes from primary, pre-antral, small antral, and large antral follicles, and protein is present in follicles (theca and granulosa cells) and oocytes of all stages including atretic follicles, but is absent from ovary epithelium (Gawriluk *et al.*, 2011). In the rat, the expression pattern of LC3A appears to be restricted to granulosa cells with weak staining in thecal cells, but no staining in oocytes (Choi *et al.*, 2010). LC3B protein is localized to the cytoplasm of oocytes and granulosa cells of all follicle stages as well as in steroidogenic cells of the corpus luteum (Ullah *et al.*, 2019; Leopardo *et al.*, 2020). Importantly, LC3B levels are significantly higher in ovaries of heat-stressed mice (Ullah *et al.*, 2019) and after exposure to FSH (Zhou *et al.*, 2017; Tang *et al.*, 2021a). Less is known about the expression of these genes in humans; however, BECN1 and LC3A are present in the KGN immortalized human granulosa cell line (Yefimova *et al.*, 2020; Li *et al.*, 2021b). Importantly, a recent *in vitro* study demonstrated that human granulosa cells can remove apoptotic oocytes by unconventional autophagy-assisted phagocytosis (Yefimova *et al.*, 2020), which may explain the expression of autophagy degradation machinery in these cells. LC3A is also expressed in cumulus cells from human cumulus–oocyte completes (COCs), with higher expression in cumulus cells classed as dysmature (Kang *et al.*, 2018). Interestingly, oocytes from COCs with dysmature cumulus cells had a much lower fertilization rate, highlighting LC3A as a possible biomarker for lower quality human cumulus cells (Kang *et al.*, 2018).

The involvement of autophagy, with or without cell death, has been reported in the establishment and maintenance of the primordial follicle reserve (Rodrigues *et al.*, 2009; Gawriluk *et al.*, 2011; Zhihan *et al.*, 2019), follicle development and atresia (Meng *et al.*, 2018), luteinization of granulosa cells and formation of corpus luteum (Tang *et al.*, 2021b), and corpus luteum regression (Choi *et al.*, 2011). Expression or activation of autophagy-related proteins is most evident in high stress conditions (such as starvation, heat, and hypoxia) and can suppress apoptotic signalling in the oocyte and/or granulosa cells in the ovary, promoting the survival of oocytes and follicles, and thereby ensuring fertility (Gannon *et al.*, 2012; Hale *et al.*, 2017; Watanabe and Kimura, 2018). Consistent with the concept that autophagy plays important survival roles in the ovary, deletion of the autophagy induction gene *Atg7* in female mice leads to excessive germ cell loss during follicle formation, reduced ovarian reserve, and subfertility (Song *et al.*, 2015). It has been proposed that autophagy protects immature oocytes from elimination by apoptosis, under starvation conditions (Song *et al.*, 2015; Wang *et al.*, 2017). Moreover, loss of function mutations in ATG7 and ATG9A are associated with autophagy impairment and ovarian failure in women (Delcour *et al.*, 2019). On the other hand, elevated expression of autophagy-related genes within granulosa cells appears to be important for the normal and insult-induced atresia of antral and pre-ovulatory follicles (Shen *et al.*, 2017, 2018; Gioia *et al.*, 2019; Ma *et al.*, 2019; Bhardwaj *et al.*, 2022), but excessive or unregulated autophagy may be pathogenic. For example, increased autophagy has been observed in ovaries from women with PCOS (Li *et al.*, 2018; Kumariya *et al.*, 2021; Xie *et al.*, 2021), which may indicate an important role for autophagy in ovarian homeostasis.

As a final note, in mature oocytes, BECN1—a key regulator of autophagosome formation and membrane trafficking—may also regulate chromosome segregation and cytokinesis during the last stages of meiosis, independent of its role in the autophagy pathway (You *et al.*, 2016). Thus, it is important to consider that expression of proteins associated with autophagy does not necessarily imply autophagy is occurring.

#### Oxidative stress, necroptosis, and pyroptosis

ROS contribute to the physiological functions of follicles and human granulosa cells (Saller *et al.*, 2012). Indeed, ROS are needed for various processes within the ovary, including ovulation (Shkolnik *et al.*, 2011; Wang *et al.*, 2022). However, accumulation of ROS from physiological stress (i.e. release of cortisol) or exposure to environmental and/or endogenous toxins can trigger various regulated cell death pathways and follicle atresia. Besides apoptosis and autophagy, necroptosis of granulosa cells and oocytes has been reported in the ovary in response to ROS accumulation. In studies performed *in vitro*, serum starving human granulosa cells causes generation of ROS and induces both necroptosis and apoptosis (Tsui *et al.*, 2017). Indeed, mediators of necroptosis, such as phosphorylated MLKL, RIPK1, and RIPK3 proteins, are readily detected in the granulosa cells of pre-antral and antral follicles in macaque ovaries and human corpora lutea (Blohberger *et al.*, 2015; Du *et al.*, 2018). Moreover, a recent publication identified elevated gene expression of RIPK1 and RIPK3 in atretic, but not healthy, bovine follicles (McEvoy *et al.*, 2021). Collectively, these data suggest that necroptosis, in concert with apoptosis and autophagy, may play a role in regulating late-stage follicle atresia, but limited mechanistic information is available.

In the ovary, granulosa cells of antral follicles are producers and targets of acetylcholine (ACh) (Mayerhofer *et al.*, 2006). ACh is an important neurotransmitter that has been implicated in the regulation of cell viability, proliferation, gap junctional communication and intracellular calcium levels, as well as expression of transcription factors (Fritz *et al.*, 2001, 2002; Kunz *et al.*, 2002; Traut *et al.*, 2009). Two esterases cleave and inactivate ACh—butyrylcholinesterase and acetylcholinesterase (AChE), with several splice variants of AChE (e.g. AChE-E, -S, and -R)—which results in isoforms that differ in subcellular localization and enzyme activity (Meshorer and Soreq, 2006). Importantly, the expression of splice variant AChE-R increases in response to oxidative stress (Härtl *et al.*, 2011; Zimmermann, 2013) and circulating levels also increase with age in humans (Sklan *et al.*, 2004). AChE-R can induce RIPK1-/MLKL-dependent necroptosis of granulosa cells (Blohberger *et al.*, 2015), which can be blocked using key inhibitors of necroptosis (e.g. the RIPK1 inhibitor necrostatin-1 and MLKL-blocker necrosulfonamide) (Blohberger *et al.*, 2015; Du *et al.*, 2018). Locally inhibiting AChE using Huperzine A, via intrabursal injection, *in vivo* for 4 weeks in rats significantly increased the number of pre-antral follicles, corpora lutea, and pup numbers, highlighting an important role for ACh in follicular development and ovulation (Urra *et al.*, 2016). Blocking the breakdown of ACh by inhibiting AChE (using Huperzine A) or interfering with necroptosis (necrostatin-1) did not improve follicle survival, but did promote oocyte development and growth of macaque follicles from the pre-antral to the small antral stage, and increased follicle granulosa cell number *in vitro* (Du *et al.*, 2018). These studies strongly implicate granulosa cell necroptosis as an additional regulated cell death pathway that can be utilized during follicle atresia. However, the mechanism by which AChE-R induces necroptosis in granulosa cells remains to be determined.

Interestingly, oxidative stress can also prime the NLR family pyrin domain containing 3 (NLRP3) inflammasome (Bauernfeind *et al.*, 2011; Won *et al.*, 2015), resulting in caspase-1 activation and pyroptosis (Strowig *et al.*, 2012). A recent study showed that polystyrene microplastics, which are transported to the ovary and taken in by the granulosa cells, result in the induction of NLRP3/caspase-1 signalling and pyroptosis activation in the ovary (Hou *et al.*, 2021). In addition, a recent study in dairy cows demonstrated that culturing granulosa cells in the presence of non-esterified fatty acids (NEFAs) induces oxidative stress, pyroptosis, and inflammation (Wang *et al.*, 2020). Specifically, NEFAs activate the toll-like receptor 4 (TLR4)/NF-κB pathway, increase the production of NLRP3 and caspase-1, and trigger granulosa cells to release inflammatory cytokines interleukin (IL)-1β and IL-6. Importantly, these effects were reversed when the granulosa cells were pre-treated with antioxidant *N*-acetylcysteine, validating the role of oxidative stress during NEFA-induced pyroptosis. The involvement of NEFAs in this process is particularly interesting as high levels of NEFAs are a hallmark of various metabolic diseases, including obesity, Type 2 diabetes, and ketosis in humans and animals (Baddela *et al.*, 2020). These circulating NEFAs can enter the ovary and the follicular fluid, and negatively impact on the steroidogenic functions of granulosa cells and oocyte quality (Yang *et al.*, 2012; Valckx *et al.*, 2014; Calonge *et al.*, 2018).

### Ovarian ageing

Reproductive ageing coincides with a decline in ovarian follicle number, leading to loss of fertility and endocrine function, and eventually menopause. Notably, emerging evidence indicates that the ovary naturally transitions to a low-level inflammatory microenvironment with advancing maternal age, termed ‘inflammageing’ (Lliberos *et al.*, 2021; Camaioni *et al.*, 2022; Umehara *et al.*, 2022). The NLRP3/apoptosis-associated speck-like protein (ASC) inflammasome, which activates caspase-1, appears to be central to this process, raising the possibility that pyroptosis might contribute to age-associated follicle loss. Although pyroptosis has not been directly studied during ovarian ageing, this hypothesis is supported by recent reports demonstrating that genetic loss or pharmacological inhibition of NLRP3 or ASC reduces caspase-1 levels in the ovary, increases oocyte number, and delays ovarian ageing (Lliberos *et al.*, 2020; Navarro-Pando *et al.*, 2021). Furthermore, the granulosa cells of women with POI exhibit elevated NLRP3, caspase-1, and IL-1α levels (Navarro-Pando *et al.*, 2021). Further investigations of how pyroptosis is regulated in the ovary will provide valuable therapeutic targets to potentially delay natural ovarian ageing.

Some new evidence suggests that parthanatos may also contribute to premature ovarian ageing. Cumulus granulosa cells collected from women with diminished ovarian reserve showed increased levels of nuclear purified PAR and AIF (Batnasan *et al.*, 2020), suggesting a role for PARP-dependent cell death in diminished ovarian reserve pathophysiology. Therefore, inhibition of parthanatos, amongst other regulated cell death pathways, may prove useful for patients with diminished ovarian reserve.

## Future directions

### Distinguishing between oocyte versus somatic cell death in ovarian follicles

Granulosa cells are specialized somatic cells in the ovary, vital for oocyte survival and female fertility. When follicles are formed, oocytes not surrounded by granulosa cells are eliminated. Impaired granulosa cell function also dysregulates oocyte growth and causes POI, characterized by early loss of fertility and hormone production (Uda *et al.*, 2004). Recent endeavours to derive human or mouse oocytes from induced pluripotent stem cells rely exclusively on mouse granulosa cells to support the germ cell-like cells (Sarma *et al.*, 2019; Stringer and Western, 2019). Collectively, these observations reveal that granulosa cells have unique and essential functional properties that cannot be replaced by other somatic cell types.

In response to activation signals, primordial follicles give rise to large, hormone-producing follicles, and mature ovulatory oocytes. The founding population of ∼5–8 granulosa cells present in a primordial follicle undergoes clonal divisions to eventually produce >2000 granulosa cells that support a mature oocyte (Hirshfield, 1991). After primordial follicle formation, there is no evidence of new granulosa cell formation from other somatic cell types (Zhang *et al.*, 2014). Thus, all mature granulosa cells appear to be clones of primordial follicle granulosa cells. As oocytes cannot survive without these essential granulosa cells, granulosa cell apoptosis invariably causes follicle atresia. However, the possibility that primordial follicle granulosa cells are susceptible to ageing and exogenous insults has not been investigated. Understanding the regulated cell death pathways utilized by each cell type comprising ovarian follicles is important to better understand the effects of maternal ageing, and to develop appropriate fertility preservation strategies for female cancer patients.

### Role of the ovarian environment in regulated cell death of ovarian follicles

Studies of the ovarian environment, including the stroma and other supporting somatic cell types, have emerged as a recent area of interest (Kinnear *et al.*, 2020). Some studies have established that fibrosis is one early hallmark of the aging ovarian stroma (Briley *et al.*, 2016; Amargant *et al.*, 2020; Umehara et al., 2022). It has thus been proposed that this altered microenvironment could contribute to the age-associated decline in oocyte number and/or quality. However, a direct link between the ovarian stroma and regulated cell death in ovarian follicles or other somatic cell types is lacking, and should be the focus of further investigation.

### Targeting regulated cell death pathways to protect fertility

Inhibiting key mediators of oocyte death is a promising strategy for protecting primordial follicles from anti-cancer treatment and age-mediated depletion. Such strategies require characterization of the pathways and specific mediators involved in order to identify suitable targets. Of relevance to cancer treatment, *Bax^–/–^* mice have prolonged fertility after chemotherapy (Kujjo *et al.*, 2010). Similarly, irradiated *TAp63^–/–^* (Stringer *et al.*, 2020), *Puma^–/–^* (Kerr *et al.*, 2012b), and checkpoint kinase 2 (*Chk2*)^*–/–*^ mice (Bolcun-Filas *et al.*, 2014; Rinaldi *et al.*, 2017) remain fertile after treatment with radiation or chemotherapy, unlike their wild-type counterparts, demonstrating the potential of this strategy for fertility preservation. Translation of this information is currently limited by the availability of effective small-molecule inhibitors to these proteins. However, an inhibitor of CHK2 does exist, and its transient use reduced irradiation-mediated primordial follicle loss in mice, leading to the generation of healthy offspring (Rinaldi *et al.*, 2017).

Targeting other regulated cell death pathways, independently or in concert with apoptosis, may also provide novel fertility preservation strategies. Indeed, investigations on the use of AChE and necroptosis inhibitors to improve folliculogenesis (Urra *et al.*, 2016; Du *et al.*, 2018), or inflammasome inhibitors to delay ovarian ageing (Navarro-Pando *et al.*, 2021; Umehara *et al.*, 2022), are exciting potential avenues for fertility preservation. However, more studies are needed to understand when and how these pathways are activated in the ovary, in order to determine the best timing and suitability of these therapies.

## Conclusion

The contribution of apoptosis to the processes of follicle formation during foetal development and around birth, as well as throughout other life stages, is well defined. However, here, we highlight substantial gaps in knowledge of the localization, activation, and contribution of key players from other types of regulated cell death pathways in ovarian follicles. Understanding the relative importance of all the different regulated cell death pathways, and how they are activated in both granulosa cells and oocytes, is vital to identify other potential therapeutic targets for fertility preservation strategies.

## Data Availability

No new data were generated or analysed in support of this research.
